# Sudden bilateral vision loss: a case report of frosted branch angiitis following pentavalent vaccination in a 2-year-old boy

**DOI:** 10.1186/s12348-025-00455-z

**Published:** 2025-02-26

**Authors:** Mohammed Falah Aljasir, Dhoha Mohammed Alhamad, Shahad Salah Alsubhi, Assaf Mohammad Almalki

**Affiliations:** Ophthalmology Department, Dhahran Eye Specialist Hospital, Dharan, Saudi Arabia

**Keywords:** Frosted branch angiitis, Uveitis, Bilateral vision loss, Pentavalent vaccination, Viral infection

## Abstract

**Background:**

Frosted branch angiitis (FBA) is a rare form of retinal vasculitis that can lead to significant vision loss. This case report presents a unique case of idiopathic FBA in a 2-year-old boy following pentavalent vaccination.

**Case Presentation:**

A previously healthy 2-year-old Emirati boy presented with sudden painless bilateral vision loss for one day. His mother noted difficulty walking and a lack of interest in visual stimuli. A few days prior, the child had received a pentavalent vaccination and experienced upper respiratory tract symptoms. Ophthalmic examination revealed bilateral dilated pupils, anterior chamber inflammation, and extensive retinal vascular sheathing. Investigations were unremarkable. The patient was treated with oral Prednisolone and Acyclovir. Within two weeks, the vision improved significantly, with complete resolution of retinal vasculitis observed within one month. Over the course of one year, the patient showed no recurrence of symptoms, and only small, stable retinal scars were noted.

**Conclusion:**

This case highlights the potential association between pentavalent vaccination and the onset of idiopathic FBA. The pathophysiology behind this association remains speculative, with potential mechanisms including immune dysregulation and molecular mimicry. Prompt recognition and treatment with systemic steroids can lead to favorable outcomes, emphasizing the importance of monitoring visual symptoms in pediatric patients following vaccination.

## Introduction

Sudden visual disability in children, though uncommon, is a serious and distressing condition that requires prompt evaluation and intervention. The causes of bilateral vision loss are diverse, including traumatic injuries, infectious diseases, neurovascular events, and psychiatric conditions [1]. Frosted branch angiitis (FBA) is a rare pan-uveitic disease characterized by vasculitis affecting the entire retina. While it typically presents bilaterally, unilateral cases can also be diagnostic. The etiology of FBA is varied and may include idiopathic, traumatic, infectious, or autoimmune factors. Infectious agents linked to FBA include cytomegalovirus (CMV), acquired immunodeficiency syndrome (AIDS), herpes simplex virus (HSV), varicella-zoster virus (VZV), Epstein-Barr virus (EBV), influenza type A, tuberculosis, toxoplasmosis, glomerulonephritis, and Streptococcus. Autoimmune disorders, such as Behçet’s disease, systemic lupus erythematosus, antiphospholipid syndrome, Crohn’s disease, and Wegener’s granulomatosis, as well as malignancies like large cell lymphoma, acute lymphoblastic leukemia, and Hodgkin’s lymphoma, can also contribute [2, 3]. Therefore, early attention is crucial for identifying the specific cause [2, 4]. FBA typically occurs in individuals aged 2 to 42 years [3, 5]. It is characterized by vascular inflammation, retinal edema, vision loss, and significant retinal vascular sheathing of both arterioles and venules. Fundus examination reveals inflamed retinal vessels, which often appear like the frosted branches of a tree [3–5]. This case report presents the first known instance of idiopathic FBA in a 2-year-old child following pentavalent vaccination.

## Case Presentation

A 2-year-old Emirati boy, a heterozygous twin A, presented to the emergency department at Dhahran Eye Specialist Hospital in the Kingdom of Saudi Arabia with sudden, painless vision loss in both eyes for one day. The child’s mother reported that he had difficulty walking and showed a lack of interest in visual stimuli, which she attributed to the bilateral sudden vision loss. Ten days prior to presentation, the child had received the pentavalent vaccination in the United Arab Emirates. His past medical history was unremarkable except for a single episode of febrile seizure six months earlier. Both prenatal and natal histories were also unremarkable. Family history was negative, and he has a healthy twin sibling. A review of systems was negative, except for symptoms of a common cold, including a runny nose and diarrhea, which had been progressing over the past five days. These symptoms were associated with a low-grade fever that lasted for just one day. The mother denied any history of trauma, loss of consciousness, skin rash, mouth ulcers, or joint pain.

**Fig. 1:**
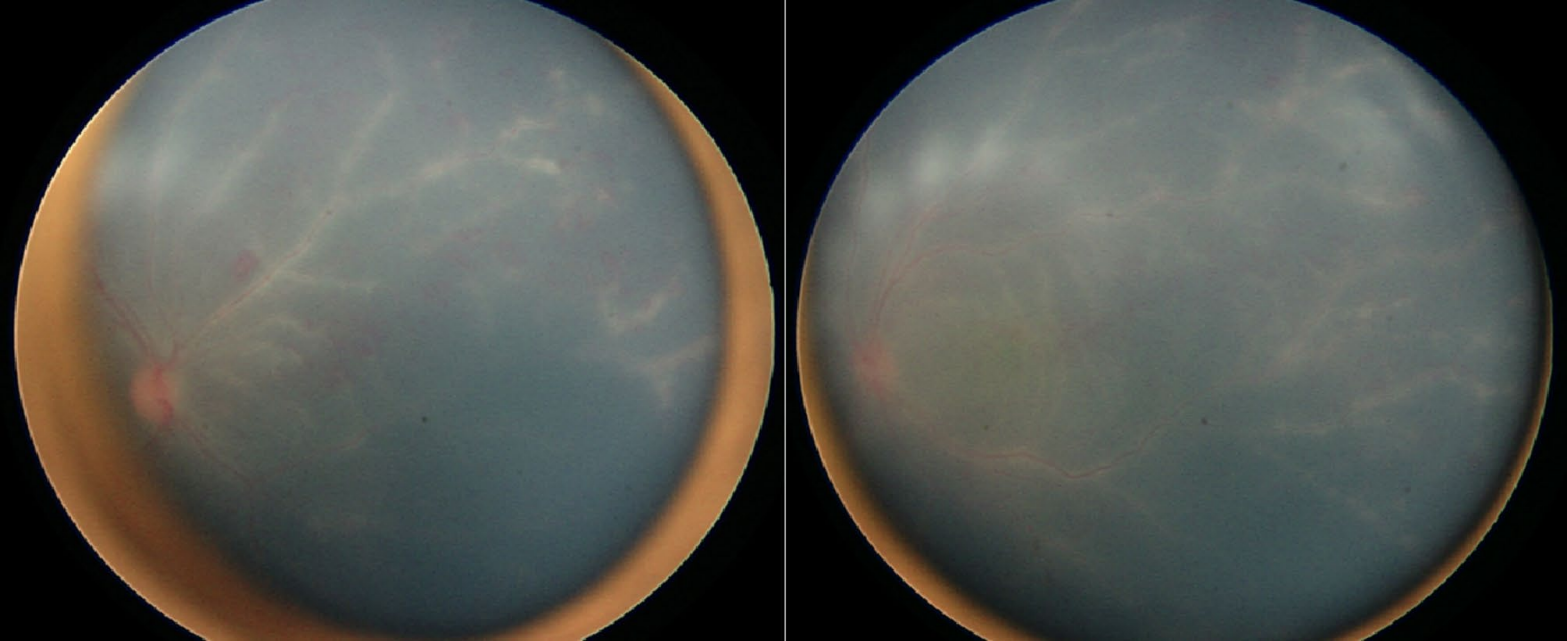
Fundus examination showing hyperemic discs, and extensive vascular sheathing in all quadrants of both eyes

**Fig. 2:**
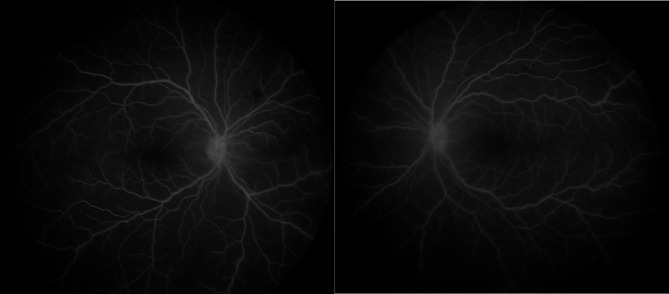
Fundus fluorescein angiography displayed normal arterial, venous, and macular perfusion with optic disc leakage

**Fig. 3:**
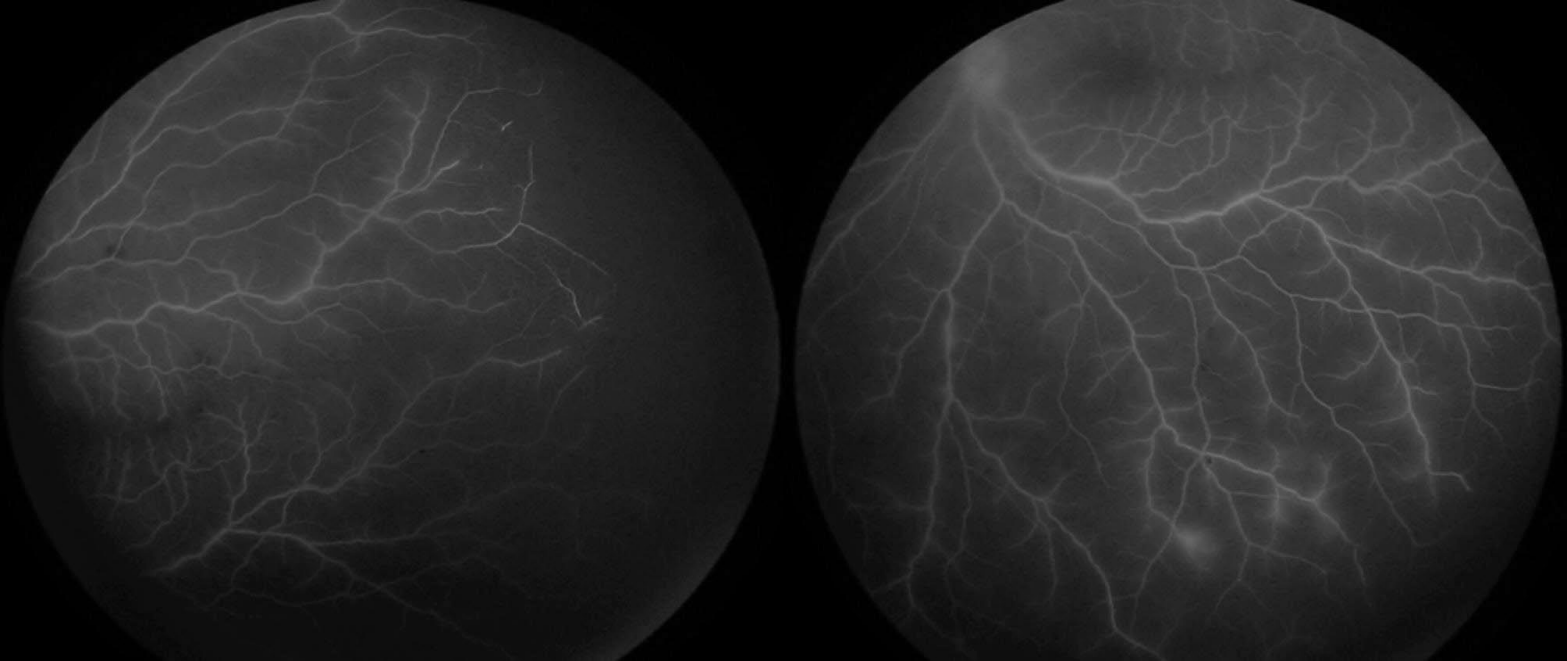
Fundus fluorescein angiography displayed diffuse vascular leakage with peripheral capillary drop out

Detailed ophthalmic examination showed that the child could not fix or follow an object. The pupils were bilaterally dilated and non-reactive. Anterior segment examination revealed + 3 pigmented cells in the anterior chamber in both eyes. Dilated fundoscopy showed hazy vitreous, hyperemic discs, and extensive vascular sheathing in all quadrants in both eyes. (Fig. 1) Fundus fluorescein angiography (FFA) demonstrated diffuse vascular leakage, including optic disc leakage (Fig. 2), as well as peripheral capillary dropout (Fig. 3). Polymerase chain reaction (PCR) analysis and culture of anterior chamber aqueous fluid for VZV, HSV 1, HSV 2, Human herpes virus 6, CMV, Haemophilus influenzae, Enterovirus, Human parechovirus, Escherichia coli K1, Listeria monocytogenes, Neisseria meningitidis (encapsulated), Streptococcus agalactiae, Streptococcus pneumoniae, Mycoplasma pneumoniae, Streptococcus pyogenes, and Cryptococcus neoformans/gattii were normal. Furthermore, thorough blood investigations including complete blood count, liver function tests, renal function tests, blood PCR of VZV, HSV and CMV, Syphilis, Human Immunodeficiency Virus (HIV), Toxoplasma latex, Purified Protein Derivative (PPD), Human Leukocyte Antigen (HLA) B27, HLA B51, Anti-Cardiolipin (IgG, IgM), Cytoplasmic Antineutrophil Cytoplasmic Antibodies (C-ANCA), Perinuclear Antineutrophil Cytoplasmic Antibodies (P-ANCA), rheumatoid factor, peripheral blood smear, chest x-ray, chest Computed Topography (CT), and brain Magnetic Resonance Imaging (MRI) were done, all of them were within normal, except high erythrocyte sedimentation rate (ESR) with a level of 26 mm/1 h.

Oral Prednisolone was prescribed, starting at 2 mg/kg/day and tapered over one month, along with Acyclovir 200 mg five times a day. Acyclovir was initially given empirically based on the possibility of a viral etiology, as suggested in the literature. However, once laboratory results indicated that the most likely etiology of FBA was autoimmune in nature, Acyclovir was discontinued, and corticosteroids remained the primary treatment. A comprehensive pediatric examination was conducted, including a thorough assessment of the child’s clinical condition. Due to the complexity of the case, the child was also evaluated by a rheumatologist, hematologist, and immunologist to rule out underlying systemic conditions. Their expert assessments confirmed the absence of systemic autoimmune or inflammatory conditions and did not recommend the use of steroid-sparing therapies.

**Fig. 4:**
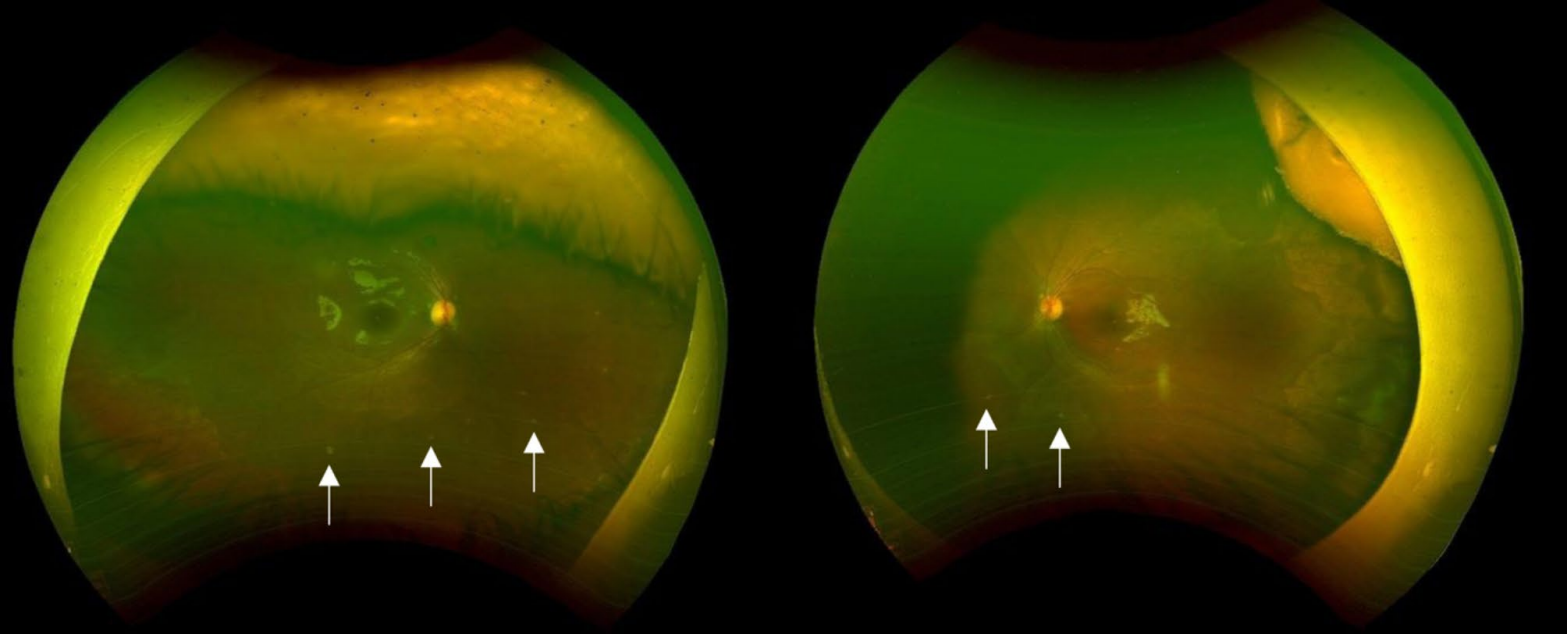
Bilateral small, diffuse, multifocal white scars in the periphery and outside the macula (arrows), with temporal peripheral retinal atrophy in both eyes

Close follow-up was initiated, with plans to taper therapy as the patient showed improvement. Two weeks after starting treatment, the patient’s vision had progressed to the ability to focus and follow with both eyes. Retinal findings demonstrated almost complete resolution of the vasculitis. One month later, fundus examination confirmed the complete resolution of vasculitis in both eyes. Over more than one year of follow-up, there was no recurrence of vasculitis, and no complications, such as retinal tears or other ocular pathologies, were observed. However, small, diffuse, multifocal white scars were noted, primarily in the periphery and outside the macula, measuring approximately 1/10th the size of the optic disc, with temporal peripheral retinal atrophy in both eyes (Fig. 4). A full field electroretinogram (ERG) response showed reduced amplitude, while visual evoked potential (VEP) showed a normal response.

## Discussions

We present a unique case of a 2-year-old child with idiopathic FBA following the pentavalent vaccination. FBA is a rare and unique type of uveitis associated with retinal vasculitis resembling the distinctive appearance of frosted branches of a tree [3–5]. The predominant reported cases were of young and healthy patients, with a peak incidence in children and third decade of life [3, 5]. Most of FBA patients presented with bilateral subacute vision loss associated with floaters and photopsia [4]. the etiologies of FBA are variable, it could be idiopathic, traumatic, infective, or post-vaccination [6–9]. 

The pentavalent vaccine, administered in the United Arab Emirates, provides protection against five serious diseases: diphtheria, pertussis, tetanus, Haemophilus influenzae type B, and hepatitis B [10]. Although the exact biological mechanism linking the pentavalent vaccine to frosted branch angiitis (FBA) remains speculative, it is noteworthy that other vaccines have been associated with autoimmune and inflammatory conditions, including FBA. Despite differences in composition and adjuvants, vaccines such as the smallpox vaccine, mRNA-1273 COVID-19 vaccine, BNT162b2 booster against SARS-CoV-2, and the diphtheria, pertussis, and tetanus (DPT) vaccine have been reported to trigger autoimmune responses, including FBA, underscoring a potential shared temporal association [6–9]. 

The DPT vaccine, which forms part of the pentavalent vaccine, has been documented in previous study as a potential trigger of FBA in a 6-year-old Japanese boy. He presented initially with visual acuity (VA) of hand motion and counting fingers in the right and left eye, respectively. Five months after administration of Steroids and Isoniazid, the VA improved to 6/60 and 6/12 in the right and left eye, respectively [9]. Similarly, our patient presented with signs and symptoms of FBA shortly after the pentavalent vaccine. The patient initially was unable to fix or follow an object. Two weeks after receiving Acyclovir and a tapering dose of oral Prednisolone, the patient’s vision progressed to the ability to focus and follow with both eyes. Regarding the other components of the pentavalent vaccine, particularly the hepatitis B virus (HBV) vaccine, they have been associated with other types of ocular diseases [11, 12]. Uveitic diseases post HBV vaccine include acute posterior multifocal placoid pigment epitheliopathy, multiple evanescent white dot syndrome, Vogt–Koyanagi–Harada syndrome, paracentral acute middle maculopathy, and posterior uveitis [12, 13]. HBV vaccination has also been reported to be associated with optic neuritis [14]. Administration of combined vaccines of diphtheria, tetanus, pertussis, and inactivated poliovirus, has been shown to be associated with optic neuritis as well [15]. 

The pathophysiology of the pentavalent vaccine activates the immune system by inducing both innate and adaptive immune responses. Although the vaccine is designed to provoke immunity against diphtheria, pertussis, tetanus, Haemophilus influenzae type B, and hepatitis B pathogens, in rare instances, it may trigger autoimmune diseases such as frosted branch angiitis (FBA). The mechanism underlying this phenomenon may involve immune dysregulation, which can result from the immune system’s response to the vaccine components. Another possible explanation is molecular mimicry, where certain proteins in the vaccine share structural similarities with self-antigens, leading the immune system to mistakenly attack the body’s own tissues, including the retinal vasculature. This immune response could lead to vasculitis in the retina, a hallmark of FBA. Additionally, adjuvants used in vaccines play a role in enhancing the immune response by stimulating innate immunity. This can lead to an overactive inflammatory response, involving the activation of autoreactive T-cells and the production of pro-inflammatory cytokines, such as interleukins (e.g., IL-6) and tumor necrosis factor (TNF-α). These cytokines may contribute to endothelial damage in the retinal blood vessels, exacerbating the autoimmune response and leading to the development of FBA. While the exact pathophysiological connection between the pentavalent vaccine and FBA remains unclear, these immune mechanisms are considered likely contributors to the rare development of autoimmune complications like FBA following vaccination [16].

Other etiologies of FBA in the pediatric population include infectious diseases such as upper respiratory tract infection, CMV, VZV, pneumonia, COVID-19, EBV, toxoplasmosis, mumps, and HIV [3, 17–24]. Many cases remain of unknown etiology [3, 25–27]. Some cases have been attributed to autoimmune diseases like systemic juvenile idiopathic arthritis, SLE, and Behçet disease [3, 4, 28, 29]. A few cases presented with headache only without a specific known etiology, while others presented predominantly with fever. Notably, one case was associated with fever and sepsis, and another child presented with fever and rash only [3]. Additionally, a single case of a child with Langerhans cell histiocytosis on clofarabine has been reported [30]. Given the wide range of potential causes, several differential diagnoses were considered for our patient. Laboratory tests ruled out associations with syphilitic, herpetic, and tubercular infections. Ophthalmic viral diseases were excluded through PCR and other investigations. The patient’s heterozygous twin was healthy, effectively ruling out genetic factors. While the exact cause remains indefinite, idiopathic FBA may have been triggered by an upper respiratory tract infection (URTI) or the pentavalent vaccination. Additionally, the child exhibited no systemic or ophthalmic signs or symptoms indicative of other etiologies.

Treatment of FBA varies depending on the etiology. Infectious causes are treated with antimicrobial therapy, while inflammatory etiologies are resolved with anti-inflammatory agents. Most of FBA patients treated with systemic steroids showed rapid resolution of symptoms with good visual recovery. Acyclovir has been given in a minority of cases [3]. The management of the four reported cases of FBA secondary to vaccination included: systemic steroids for one patient; a combination of systemic steroids and antimicrobial therapy (Isoniazid) or antiviral therapy (Acyclovir) for two patients; and systemic steroids followed by vitrectomy for the last patient, who had an uneventful recurrent disease [6–9]. Our patient’s young age suggested the use of steroid-sparing agents; however, multidisciplinary evaluations confirmed the absence of systemic involvement, justifying the use of systemic Corticosteroids alone. Acyclovir was initially administered empirically based on a potential viral etiology suggested in the literature but was discontinued when laboratory findings indicated an autoimmune origin, leaving Corticosteroids as the primary treatment.

The prognosis of FBA varies considerably, with recovery times and outcomes differing across reported cases. In the majority of cases, good visual outcomes are achieved within a month, while a minority experience prolonged recovery. Complications reported in the literature include macular scarring, retinal vein or artery occlusion, macular epiretinal membrane formation, diffuse retinal fibrosis, retinal tear formation, vitreous hemorrhage, optic disc atrophy, and peripheral atrophic retinal lesions [3]. Our patient showed progressive improvement within two weeks and complete resolution of vasculitis in less than one month. The only residual finding was small, multifocal white scars in both eyes, predominantly in the periphery, measuring approximately 1/10th the size of the optic disc. Scarring can be associated with various etiologies of FBA, including idiopathic causes, infectious conditions such as CMV, and non-infectious diseases like Behçet’s disease. Therefore, it is not indicative of any specific etiology [3, 17, 31]. No complications or disease recurrence were observed during more than one year of follow-up, which supports the variability in outcomes and underscores the importance of close monitoring.

The limitations of this case report include the absence of a COVID-19 test, as well as the lack of investigation into less common viral etiologies such as parainfluenza and parvovirus B19. Additionally, inflammatory markers like IL-6 and TNF-α were not assessed. As this is a single case study, the causal link remains inconclusive; this idiopathic FBA could be secondary to the pentavalent vaccination or an upper respiratory tract infection. Further research is needed to better understand the link and mechanism of action between FBA and vaccination, particularly with the pentavalent vaccine.

## Conclusion

This case report describes a rare occurrence of frosted branch angiitis (FBA) in a 2-year-old child following pentavalent vaccination, a first of its kind to our knowledge. The potential pathophysiological link between the pentavalent vaccine and FBA remains unclear, though immune dysregulation and molecular mimicry are speculated to play a role in triggering autoimmune conditions such as FBA. This case highlights the critical need for a comprehensive differential diagnosis. In this instance, the child responded favorably to corticosteroid therapy, experiencing rapid improvement and complete recovery of vision within less than a month. Follow-up over a year revealed only minor retinal scars, underscoring the effectiveness of prompt treatment in achieving a favorable outcome.

## Data Availability

No datasets were generated or analysed during the current study.

## Abbreviations

FBA: Frosted Branch Angiitis
CMV: Cytomegalovirus
AIDS: Acquired Immunodeficiency Syndrome
HSV: Herpes Simplex Virus
VZV: Varicella-Zoster Virus
EBV: Epstein-Barr Virus
FFA: Fundus Fluoresceine Angiography
PCR: Polymerase Chain Reaction
HIV: Human Immunodeficiency Virus
PPD: Purified Protein Derivative
HLA: Human Leukocyte Antigen
C-ANCA: Cytoplasmic Antineutrophil Cytoplasmic Antibodies
P-ANCA: Perinuclear Antineutrophil Cytoplasmic Antibodies
MRI: Magnetic Resonance Imaging
ESR: Erythrocyte Sedimentation Rate
ERG: Electroretinogram
VEP: Visual Evoked Potential
mMRNA-1273: Messenger RNA-1273 COVID-19 Vaccine
BNT162b2: Pfizer-BioNTech COVID-19 Vaccine
VA: Visual Acuity
HBV: Hepatitis B Virus
